# Identification of the High-Affinity Potassium Transporter Gene Family in Perennial Ryegrass (*Lolium perenne*) and Its Potential Role in Salt Stress

**DOI:** 10.3390/genes16111341

**Published:** 2025-11-07

**Authors:** Xin Song, Xixiong Hong, Huilan Zeng, Peipei Su, Minshan Sun

**Affiliations:** 1 School of Life Science and Environmental Resources, Yichun University, Yichun 336000, China; 207209@jxycu.edu.cn (X.H.); zenghl@jxycu.edu.cn (H.Z.); ppsu886@163.com (P.S.); 2 College of Plant Protection, Henan Agricultural University, Zhengzhou 450002, China

**Keywords:** high-affinity potassium transporter (HKT), *Lolium perenne*, abiotic stress, salt tolerance

## Abstract

**Background:** Perennial ryegrass (*Lolium perenne* L.), a widely cultivated turfgrass and forage species in Europe and North America, exhibits rapid growth and notable salt tolerance. The *high-affinity potassium transporter* (*HKT*) gene family has been implicated in salt stress responses across multiple plant species. However, whether the salt tolerance of *L. perenne* is closely associated with its HKT gene family remains unclear. **Methods and Results:** In this study, we systematically identified HKT family members in the *L. perenne* genome. Five *HKT* genes were identified and classified into three subfamilies. Among these, *LpHKT1a–c* exhibited canonical class I features with a conserved serine (S) residue in the P1 domain, whereas *LpHKT2* belonged to class II, characterized by a glycine (G) residue in the same domain. Notably, LpHKT3 formed a distinct subfamily with a truncated structure and divergent P1/P2 domains, suggesting potential non-canonical functions. *LpHKT1a* likely lacked the P4 domain. Promoter analysis revealed that all five *LpHKT* genes contain multiple stress-related *cis*-acting elements. Real-time quantitative reverse transcription polymerase chain reaction results showed that *LpHKT1b/c* and *LpHKT2* were highly expressed in both roots and leaves. Under low-concentration NaCl stress (25 mM), the expression of these three genes significantly increased by 8- to 12-fold at 6–12 h post-treatment (vs. control). Ion accumulation analysis demonstrated a rapid increase in Na^+^ levels following NaCl treatment, whereas K^+^ concentrations initially remained stable but significantly increased after 24 h. **Conclusions:** Combined with the cellular localization of *LpHKT1c* predominantly in the xylem, these findings suggest that *LpHKT* genes may be involved in Na^+^ and K^+^ transport in roots. This study represents the first genome-wide identification of the *HKT* gene family in *L. perenne*, providing critical insights into the molecular mechanisms underlying its salt tolerance and offering valuable genetic resources for molecular breeding aimed at enhancing stress resilience.

## 1. Introduction

Soil salinization is a major abiotic stress factor limiting global agricultural production, directly affecting plant ion homeostasis and growth [1,2]. Plants maintain ionic equilibrium and enhance salt tolerance through the precise regulation of Na^+^/K^+^ dynamics by high-affinity potassium transporters (HKTs), which play a central role in ion homeostasis [3,4]. Studies have shown that HKT family members contribute to critical processes such as root salt exclusion and xylem ion unloading by selectively transporting Na^+^ or symporting Na^+^/K^+^ [5]. For instance, AtHKT1;1 in *Arabidopsis thaliana* reduces Na^+^ accumulation in shoots by mediating Na^+^ unloading in xylem parenchyma cells, thereby improving salt tolerance [6]. Similarly, OsHKT1;5 in rice (*Oryza sativa*) regulates Na^+^ compartmentalization in vascular tissues, leading to varietal differences in salt tolerance [7]. Additionally, *HKT* genes are implicated in heavy metal stress responses; for example, *ZmHKT1c* in maize (*Zea mays*) alleviates cadmium toxicity by modulating K^+^ homeostasis [8]. Recent studies also implicate K transporters in heavy metal detoxification, HKT1 and HKT5 in sweet potato modulates K^+^ homeostasis to alleviate cadmium toxicity [9].These findings highlight the multifunctional roles of *HKT* genes in plant stress adaptation; however, their regulatory networks in non-model species, particularly forage grasses, remain poorly understood.

Despite extensive characterization of *HKT* genes in model plants, research on monocot forage species such as perennial ryegrass (*L. perenne*) remains limited. As a globally cultivated high-quality forage, *L. perenne* holds significant potential for saline–alkali soil rehabilitation and sustainable agriculture [10,11]. However, salt stress severely reduces its biomass and quality, threatening the stability of pasture production systems [12]. Although phenotypic variations in salt tolerance have been reported in *L. perenne* [2,13,14,15,16], the molecular mechanisms and regulatory networks of *HKT* genes lack systematic investigation. The HKT proteins share four transmembrane domain-pore domain-transmembrane domain units’ characteristic structure (P1–P4) [17]. The HKT gene family is classified into two subclasses based on key residue differences in transmembrane domains: class I (S-G-G-G, Na^+^ uniporters) and class II (G-G-G-G, Na^+^/K^+^ symporters). In monocots, these subclasses often coexist and function complementarily. For example, rice OsHKT2;1 (class II) mediates high-affinity K^+^ uptake, whereas OsHKT1;5 (class I) specifically regulates Na^+^ transport [7]. Recent studies reveal that wheat TaHKT1;5 and rice OsHKT1;5 enhance salt tolerance by unloading Na^+^ from the xylem, whereas barley HvHKT1;5 unexpectedly promotes Na^+^ shootward transport, indicating functional divergence of HKTs within cereals [18,19]. Notably, the functional divergence of *HKT* genes is closely linked to structural features; for instance, maize *HKT1;1* gene displays complex splicing patterns that confer salt tolerance in transgenic tobacco plants, suggesting diversified regulatory mechanisms [20]. However, whether *L. perenne HKT* genes follow similar classification principles and whether structural variations drive environmental adaptation remain unresolved.

To address these gaps, this study systematically analyzed the *HKT* gene family and characterized its expression profile in salt-tolerant and salt-sensitive cultivars of *L. perenne*. By integrating bioinformatics, molecular biology, and physiological analyses, it aimed to elucidate the evolutionary classification, structural features, and dynamic expression patterns of *HKT* genes under salt stress. The findings will advance our understanding of the *HKT* gene family in *L. perenne* and provide critical genetic resources for breeding salt-tolerant varieties.

## 2. Materials and Methods

### 2.1. Plant Materials and Growth Conditions

*L. perenne* cultivars Inspire and Catalina were used in this study. Inspire was highly susceptible to salt, whereas Catalina was highly resistant. Seeds were sown in sand and cultured at a constant temperature of 20 °C/15 °C (12 h light/12 h dark) in a growth chamber with a light intensity of 400 µmol·m^−2^·s^−1^. During the growth period, 50 mL of 1/2 Hoagland nutrient solution was applied to each flowerpot daily. All plants were regularly trimmed to a height of 5–6 cm every week to stimulate tillering.

### 2.2. Salt Stress Treatments

The cultivars Inspire and Catalina were further cultured for 4 weeks and then treated with 25 mM and 100 mM NaCl solutions, respectively. Salt treatments were applied by irrigating pots with NaCl solutions; plants remained in original pots to avoid transplant stress. Control groups received equivalent volumes of nutrient solution without NaCl. Fresh root, stem, and leaf samples were collected at 0, 6, 12, and 24 h post-treatment (hpt) and were immediately stored in liquid nitrogen for further analysis.

### 2.3. Ion Accumulation Analysis

All plants were continuously cultured for 4 weeks under the same conditions as described above, after which the seedlings were treated with 25 mM NaCl. In the 100 mM NaCl treatment group, the salt concentration was gradually increased by 25 mM every 24 h until reaching the final concentration. Afterwards, all plants (including the control group) were pruned to a height of 5–6 cm and then treated with 25 mM and 100 mM NaCl for 15 days as mentioned above. Samples were dried at 65 °C for 2 days and dried powdered samples (50 mg) were digested with 18 M H_2_SO_4_ (5 mL) at 200 °C for 30 min. After cooling, 30% H_2_O_2_ (5 mL) was added, followed by reheating (200 °C, 30 min) until clarification. Digestates were diluted to 50 mL and analyzed for K^+^/Na^+^ using ICP-AES (ICP 9820, Shimadzu, Columbia, MD, USA). Ion concentration data are expressed as mean ± SD. Differences between cultivars or treatments were analyzed by two-way ANOVA.

### 2.4. Identification and Annotation of HKT Family Genes

The reported *HKT* genes of *A. thaliana* (version: TAIR10, https://www.arabidopsis.org/), *O. sativa* (version: 7.0, http://rapdb.dna.affrc.go.jp/download/irgsp1.html (accessed on 9 September 2025) GCF_001433935.1), and *Z. mays* (GCA_000005005.4, https://www.ncbi.nlm.nih.gov/), including AtHKT1, OsHKT1–OsHKT9, ZmHKT1c, and ZmHKT2a, were downloaded based on previous reports [1,8,21]. *L. perenne* genome data (version 2.0) were downloaded from NCBI (GCF_019359855.2, https://ftp.ncbi.nlm.nih.gov/). To comprehensively identify HKT homologs in *L. perenne*, a dual approach was employed. First, a local BLASTp 2.2.30 search was conducted using known HKT protein sequences from *A. thaliana*, *O. sativa*, and *Z. mays* as queries against the *L. perenne* proteome. Second, a Hidden Markov Model (HMM) profile for the TrkH domain (PF02386) was used to scan the same proteome dataset using HMMER 3.0 software with an E-value threshold of <1 × 10^−5^ [22]. The resulting candidates were further verified using Pfam, NCBI Conserved Domain Database, and SMART tools [23,24,25]. Pseudogene screening was performed using CPC2 (default parameters) to confirm protein-coding potential of all candidates [26].

### 2.5. Phylogenetic and Sequence Analysis of Genes of the HKT Family

BioEdit 7.2.5 was used to perform multiple sequence alignments of the HKT proteins identified above. A phylogenetic tree was constructed using MEGA 7.0 software with the Neighbor-Joining method based on the Poisson correction model and 1000 bootstrap replicates [27]. A schematic diagram of the gene structure was generated using TBtools 1.0 [28].

### 2.6. Structural Analysis of Conserved Motifs and Genes of the HKT Family

Conserved protein motifs were identified using the Multiple Em for Motif Elicitation program (http://meme-suite.org/) with default parameters. The 1500 bp promoter region sequence of each *HKT* gene was used to analyze *cis*-acting elements using PlantCARE (http://bioinformatics.psb.ugent.be/webtools/plantcare/html/, accessed on 9 September 2025) [29].

### 2.7. Quantitative Reverse Transcription Polymerase Chain Reaction (qRT-PCR) Analysis of HKT Genes

Total RNA was extracted following the instructions of the Direct-zol™ RNA MiniPrep Kit (Zymo Research Corporation, Irvine, CA, USA). Freshly isolated RNA was reverse-transcribed into cDNA using the iScript™ cDNA Synthesis Kit (Bio-Rad, Hercules, CA, USA) according to the manufacturer’s protocol. *LpEF1a* was used as the internal reference gene. The primers used for all detected genes are listed in Supplementary Table S1. All qRT-PCR assays passed single-peak melt curves, and electrophoretic confirmation of specific amplicons. Three biological replicates, each with six technical replicates, were included. Relative gene expression levels were calculated using the 2^−ΔΔCt^ method. Data are presented as mean ± standard deviation (SD) of three biological replicates. Statistical significance was determined using Student’s *t*-test or two-way ANOVA, with *p* < 0.05 considered significant.

### 2.8. In Situ Hybridization Assay of LpHKT1c

Fresh roots from *L. perenne* treated with 50 mM NaCl for 8 h were fixed in pre-chilled FAA solution (63% ethanol, 5% acetic acid, 2% formaldehyde, and 30% RNase-free water) for 12 h, followed by sequential washes with 63% ethanol/5% acetic acid and 1× PBS. Tissues were embedded in 5% low-melting-point agarose (in 1× PBS), frozen in liquid nitrogen, and sectioned at 50 μm using a Leica microtome (Wetzlar, Giessen, Germany). Sections were treated with DNase I (Takara, Osaka, Japan) to remove genomic DNA, reverse-transcribed using the PrimeScript™ cDNA Synthesis Kit (Takara), and subjected to PCR using DIG-11-dUTP-labeled probes. After PCR, sections were blocked with 0.1% BSA, incubated with anti-digoxin-alkaline phosphatase antibody (1:500), and stained with BM purple AP substrate for microscopic imaging.

## 3. Results

### 3.1. Identification of the HKT Gene Family in L. perenne

In this study, five HKT family members were identified in *L. perenne*. Based on their homology to previously characterized genes, these members were designated as *LpHKT1a–c*, *LpHKT2*, and *LpHKT3* (Table 1). The encoded amino acid sequences ranged in length from 257 to 1278 residues. Specifically, LpHKT1a–c were approximately 500 amino acids in length, while LpHKT2 was the longest (1278 aa) and LpHKT3 the shortest (257 aa). The molecular weights of the proteins ranged from 28.5 to 142.1 kDa, with theoretical isoelectric points ranging from 8.18 to 9.95. HMMER predictions revealed that LpHKT1a–c and LpHKT3 each contained a single canonical TrkH domain, whereas four TrkH domains were identified in LpHKT2. CPC2 analysis confirmed all five *LpHKT* genes possess protein-coding potential (Supplementary Table S2).

### 3.2. Multiple Sequence Alignment of HKT Proteins

**Figure 1:**
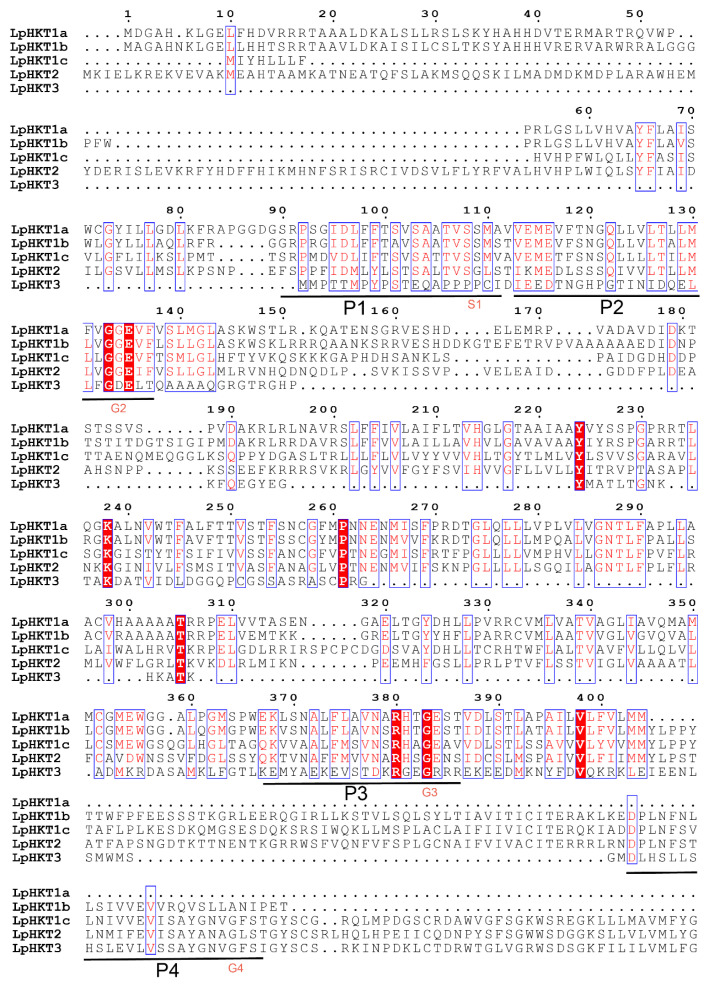
Sequence alignment of 5 HKT proteins of *Lolium perenne*. The sequences were aligned using ClustalW. The conserved protein motif is shown in figure, horizontal line shown the four pore motifs (P1–P4). The S1 and G2–4 amino acid residue under horizontal line indicated conservation pore-forming regions critical residue, respectively.

Multiple sequence alignment of the five LpHKT proteins revealed that their sequences generally conform to the four conserved pore domains (P1–P4). HKTs are categorized into class I (S-G-G-G, functioning as sodium uniporters) and class II (G-G-G-G, acting as Na^+^/K^+^ symporters) based on the residue in the first pore domain (P1). The P1 domains of *L. perenne* LpHKT1a–c contained a conserved serine residue (S), classifying them as class I members, whereas LpHKT2 exhibited a glycine residue (G) in P1, placing it in class II. This observation indicates that *L. perenne*, as a monocotyledonous species, harbors both class I and class II HKT family members. The P1–P3 domains displayed relatively high conservation across all members. However, LpHKT3 showed substantial divergence in its P1 and P2 domains, with only the P3 and P4 domains retaining conserved key amino acids, suggesting a non-canonical regulatory role requiring validation. Notably, LpHKT1a lacked the P4 domain entirely, retaining only P1–P3 (Figure 1).

### 3.3. Phylogenetic Tree of LpHKT Genes

**Figure 2:**
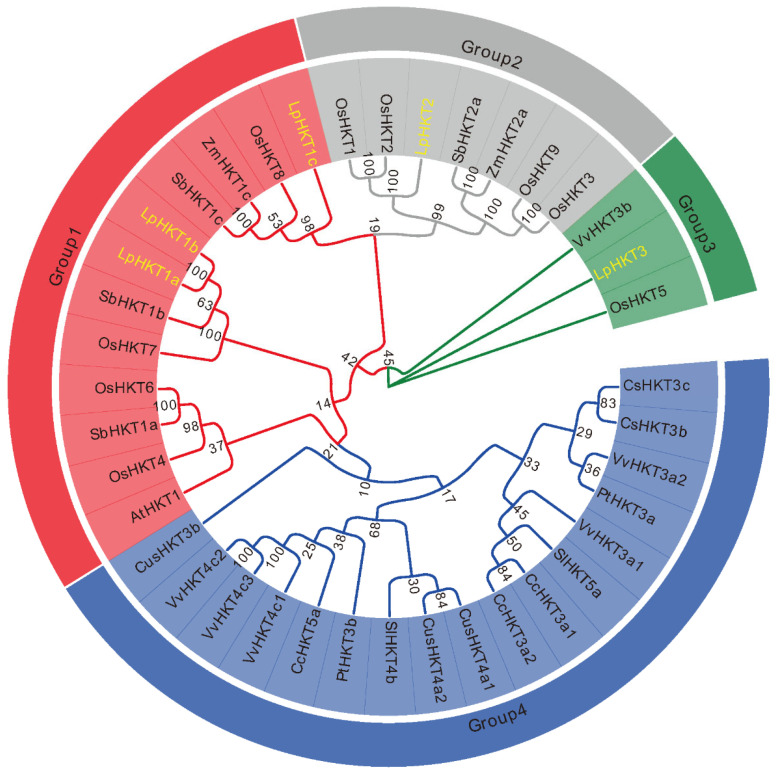
Phylogenetic analysis of the HKT proteins. The evolutionary history was inferred using the Neighbor-Joining method. yellow color: the HKT proteins of *L. perenne*. The bootstrap consensus tree inferred from 1000 replicates is taken to represent the evolutionary history of the analyzed taxa. The percentage of replicate trees in which the associated taxa clustered together in the bootstrap test (1000 replicates) are shown next to the branches. Evolutionary analyses were conducted in MEGA 7.0. CsHKT3c:XP_006474312; CsHKT3b: XP_006474646; SbHKT1c: XP_002457736; SbHKT1a: XP_002451638; SbHKT1b: KXG27071; SbHKT2a: XP_002438960; SlHKT5a: NP_001289833; SlHKT4b: NP_001295273; ZmHKT1c: DAA54361; ZmHKT2a: XP_008645031; CcHKT3a1: CDP16110; CcHKT3a2: CDP17691; CcHKT5a: CDP16112; VvHKT4c1: CBI40134, VvHKT3a2: CBI40144; VvHKT4c3: CBI40132; VvHKT4c2: CBI40133; VvHKT3a1: CBI40140; VvHKT3b: CBI40137; CusHKT4a1: XP_004150748; CusHKT3b: XP_004149678; CusHKT4a2: XP_004150749; PtHKT3b: XP_011017602; PtHKT3a: XP_002325229; AtHKT1: NP_567354; ZmHKT1c: DAA54361; ZmHKT2a: XP_008645031; OsHKT1: BAB61789; OsHKT2: BAB61791; OsHKT3: CAD37187; OsHKT4: CAD37183; OsHKT5: ATU90103; OsHKT6: CAD37185; OsHKT7: BAG98930; OsHKT8: ATU90106; OsHKT9: CAD37199.

To elucidate the evolutionary relationships among HKT family members, a phylogenetic tree was constructed using LpHKT proteins from *Arabidopsis*, *Z. mays*, *O. sativa*, *L. perenne*, *Citrus sinensis*, *Sorghum bicolor*, *Populus trichocarpa*, *Solanum lycopersicum*, *Coffea canephora*, *Vitis vinifera*, and *Cucumis sativus* [8]. The results revealed that these proteins clustered into four distinct subfamilies. LpHKT1a–c grouped within the group 1 subfamily. LpHKT2, together with OsHKT1–OsHKT3, OsHKT9, ZmHKT2a and so on, formed the group 2 subfamily. Notably, LpHKT3 exhibited close evolutionary ties to OsHKT5 from rice, defining group 3 subfamily. Other members formed the group 4 subfamily (Figure 2).

### 3.4. Gene Structures and Conserved Motif Analysis of LpHKT Genes

**Figure 3:**
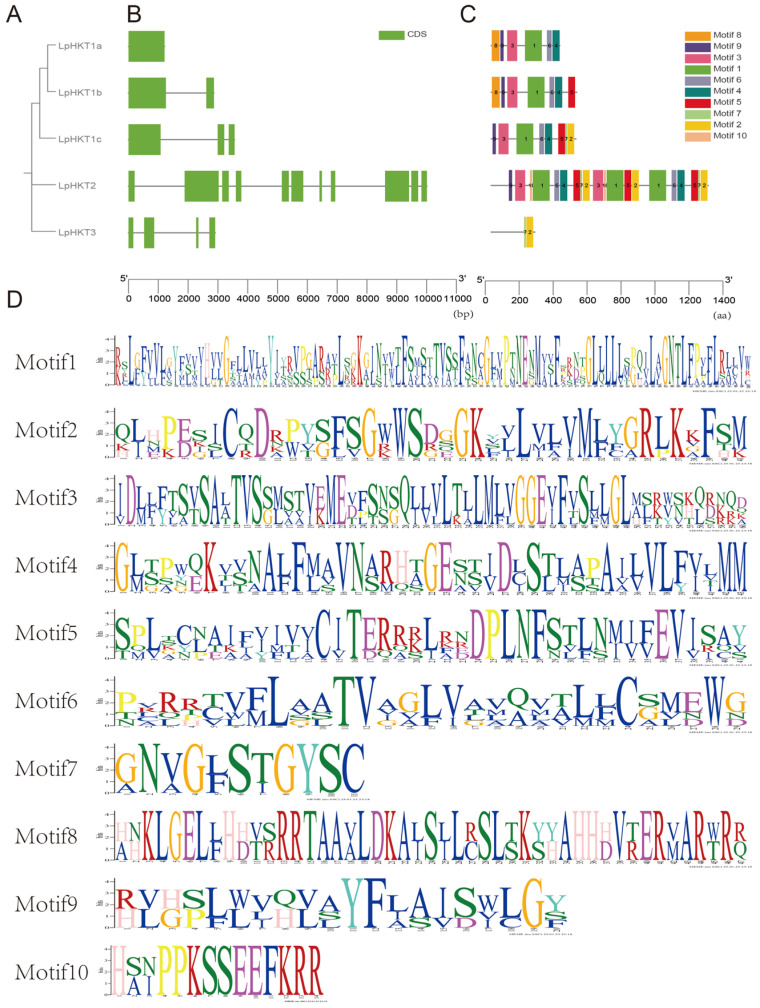
Structural and evolutionary features of *LpHKT* genes. (**A**) Phylogenetic tree showing three clades. (**B**) Exon–intron organization, with exons represented by green boxes and introns by gray lines. (**C**) Distribution of conserved motifs (1–10). Each motif is indicated with a special color. (**D**) Sequence logos of key motifs highlighting domain-specific residues.

The five *L. perenne HKT* members were classified into three distinct clades (Figure 3A). Analysis of gene structures revealed low similarity among these genes: *LpHKT1a* contained a single exon, *LpHKT1b* had two exons, *LpHKT1c* possessed three exons, *LpHKT3* comprised four exons, and *LpHKT2* exhibited the most complex structure with 11 exons (Figure 3B). Conserved motif analysis of the top 10 motifs showed that LpHKT1a–c and LpHKT2 predominantly contained motifs 1, 3, 4, 6, and 9. Additionally, LpHKT1c, LpHKT2, and LpHKT3 shared motifs 2 and 7, which are primarily located downstream of the P4 domain. Notably, LpHKT2 harbored multiple copies of motifs 1, 2, 4, 5, 6, and 7, potentially linked to sequence duplication events, although further investigation is required to confirm this hypothesis (Figure 3C). Functional annotation of motifs indicated that motif 3 corresponds to the canonical features of the P1 and P2 domains, motif 4 aligns with the P3 domain, and motif 1 spans the interdomain region between P2 and P3 (Figure 3D).

### 3.5. Promoter Cis-Acting Element Analysis of LpHKT Genes

**Figure 4:**
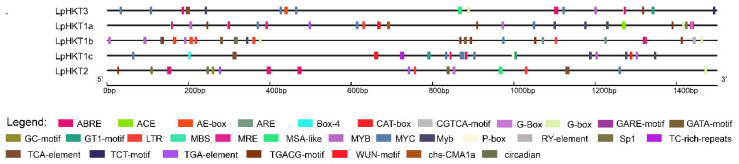
*cis*-acting elements in the promoters of *LpHKT* genes.

*Cis*-acting elements in promoters are closely associated with the regulation of gene expression. Analysis of the promoters of the five *LpHKT* genes revealed that all promoters harbored multiple types of stress-responsive *cis*-acting elements. Abiotic stress-related elements included ABRE (ABA-responsive element), MYB/MYC binding sites, and TC-rich repeats, whereas biotic stress-related elements encompassed the G-box. Multi-stress elements such as ARE (antioxidant-responsive element) and GARE (gibberellin-responsive element) were also identified. The abundance of stress-related *cis*-elements in these promoters suggests that *L. perenne HKT* genes not only play critical roles in ion transport but also contribute significantly to plant stress adaptation (Figure 4).

### 3.6. Expression Profiles of LpHKT Genes

**Figure 5:**
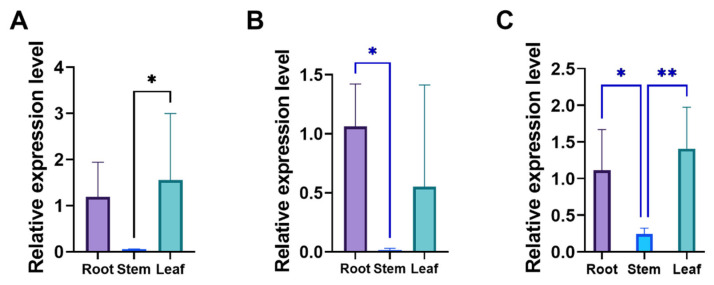
The relative expression pattern of *LpHKT* genes in perennial ryegrass. (**A**) LpHKT1b. (**B**) LpHKT1c. (**C**) LpHKT2. 4 weeks seedlings were used for testing (n = 8). All data are presented as the mean (±SD) of three independent biological determinations and were analyzed by Student’s *t*-test (* *p* < 0.05, ** *p* < 0.01).

*HKT* genes are primarily associated with ion transport and stress responses. To further investigate their tissue-specific expression patterns, qRT-PCR analysis revealed that *LpHKT1b*, *LpHKT1c*, and *LpHKT2* exhibited significantly higher expression in roots and leaves (*p* < 0.05) than in stems, respectively (Figure 5).

**Figure 6:**
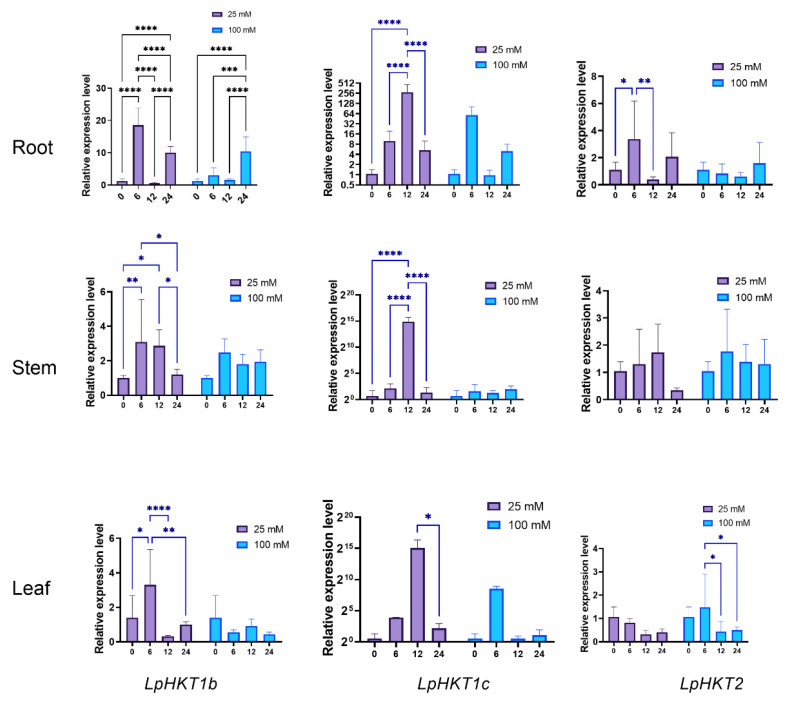
Expression patterns of *LpHKTs* under 25 mM and 100 mM NaCl solution treatment. 4 weeks seedlings were used for testing (n = 8). All data are presented as the mean (±SD) of three independent biological determinations and were analyzed by two-way ANOVA (* *p* < 0.05, ** *p* < 0.01, *** *p* < 0.001, **** *p* < 0.0001). Catalina was used for salt tress treatment.

To assess salt stress responsiveness, seedlings were treated with two NaCl concentrations. Significant expression changes were observed in root, stem, and leaf tissues under stress conditions. Specifically, the expression of *LpHKT1b*, *LpHKT1c*, and *LpHKT2* increased at 6 hpt. However, *LpHKT1b* and *LpHKT2* showed a rapid decline at 12 hpt, whereas *LpHKT1c* peaked at 12 hpt before decreasing thereafter. Notably, under high-concentration NaCl treatment, *LpHKT1b* in roots and *LpHKT2* in leaves displayed pronounced responses. Conversely, all three genes exhibited more robust transcriptional activation under low-concentration salt stress (Figure 6).

**Figure 7:**
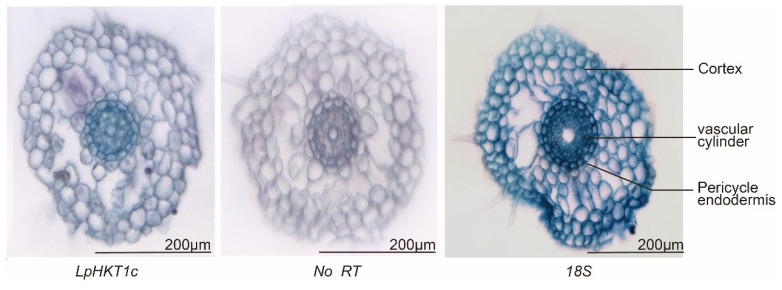
The expression tissue localization of LpHKT1c. No RT mean no signal; 18S, positive control. Scale bars: 200 μm.

Further in situ hybridization analysis revealed that *LpHKT1c* expression was predominantly localized in the vascular cylinder (Figure 7).

### 3.7. Ion Accumulation After Salt Treatment

**Figure 8:**
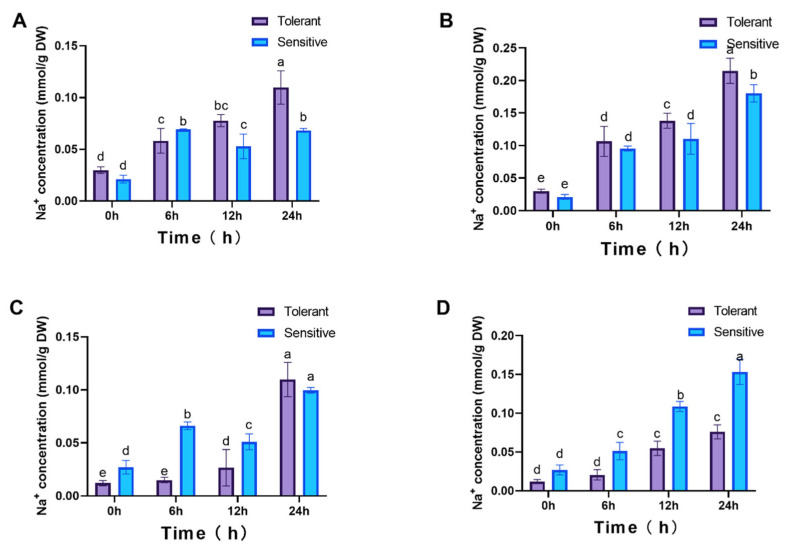
Na^+^ accumulation dynamics in plants. Roots (**A**,**B**) and leaves (**C**,**D**) under 25 mM and 100 mM NaCl treatments, respectively. 4 weeks seedlings were used for testing (n = 8). All data are presented as the mean (±SD) of three independent biological determinations and were analyzed by two-way ANOVA (lowercase letters indicate significant differences among different varieties or treatments at the same time point (*p* < 0.05)). Varieties Inspire (sensitive) and Catalina (tolerant) are used for salt treatment.

To determine the relationship between gene expression trends and ion concentrations, Na^+^ levels in roots and leaves were measured under two NaCl concentrations, which revealed notable differences between tolerant and sensitive cultivars. In roots, Na^+^ accumulation in the tolerant cultivar was markedly higher than in the sensitive one at multiple time points (*p* < 0.05, Figure 8A,B). Conversely, in leaves, Na^+^ concentrations in the tolerant cultivar remained significantly lower than those in the sensitive cultivar across all time points (Figure 8C,D).

**Figure 9:**
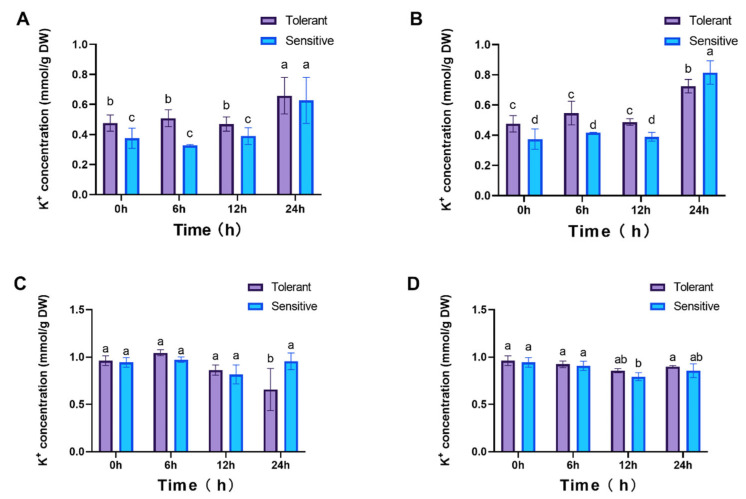
K^+^ concentrations in plants. Roots (**A**,**B**) and leaves (**C**,**D**) under 25 mM and 100 mM NaCl treatments, respectively. 4 weeks seedlings were used for testing (n = 8). All data are presented as the mean (± SD) of three independent biological determinations and were analyzed by two-way ANOVA (lowercase letters indicate significant differences among different varieties or treatments at the same time point (*p* < 0.05). Varieties inspire (sensitive) and catalina (tolerant) are used for salt treatment.

Given that the class II transporter *LpHKT2* may function as a Na^+^/K^+^ symporter, K^+^ concentrations were also examined. In roots, no significant differences in K^+^ levels were observed between cultivars from 0 to 12 hpt. However, by 24 hpt, K^+^ levels had increased significantly, with the tolerant cultivar exhibiting higher concentrations than the sensitive one during the 0–12 hpt period (Figure 9A,B). In leaves, under 25 mM NaCl treatment, K^+^ concentrations showed no significant variation except for a marked decline in the tolerant cultivar at 24 hpt. Under 100 mM NaCl, K^+^ levels in the tolerant cultivar remained stable, whereas a substantial reduction was observed in the sensitive cultivar at 12 hpt (*p* < 0.05, Figure 9C,D). These correlations suggest LpHKT involvement in Na^+^/K^+^ homeostasis, though direct transport activity requires validation.

**Figure 10:**
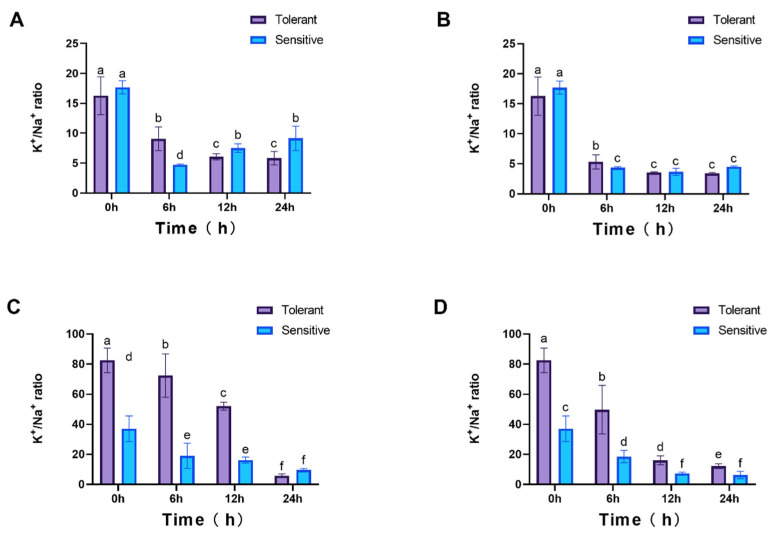
K^+^/Na^+^ ratio in plants. Roots (**A**,**B**) and leaves (**C**,**D**) under 25 mM and 100 mM NaCl treatments, respectively. 4 weeks seedlings were used for testing (n = 8). All data are presented as the mean (±SD) of three independent biological determinations and were analyzed by two-way ANOVA (lowercase letters indicate significant differences among different varieties or treatments at the same time point (*p* < 0.05)). Varieties inspire (sensitive) and catalina (tolerant) are used for salt treatment.

The K^+^/Na^+^ ratio was also analyzed under both NaCl treatments, and a significant decrease was observed following salt exposure. In roots, no initial differences (0 hpt) were detected between cultivars. However, under 25 mM NaCl, the ratio diverged significantly at multiple time points, whereas under 100 mM NaCl, differences were only evident at 6 hpt (Figure 10A,B). In leaves, the K^+^/Na^+^ ratio exhibited more pronounced fluctuations, with significant cultivar-specific differences (Figure 10C,D).

## 4. Discussion

*L. perenne* is an important turfgrass and forage species known for its strong salt tolerance. HKTs, which are closely associated with Na^+^ and K^+^ translocation, have been extensively studied in model species such as *A. thaliana* [3,4,5,6], *O. sativa* [30,31,32,33], and *Z. mays* [34,35,36,37]. This study investigated the HKT gene family in *L. perenne* to elucidate the relationship between HKT genes and salt tolerance in forage grasses.

### 4.1. Classification and Evolutionary Features of the HKT Gene Family

This study identified five HKT family members in *L. perenne*, grouped into the HKT1, HKT2, and a novel HKT3 subfamily. No pseudogenes were detected, indicating robust identification. LpHKT1a–c (class I) and LpHKT2 (class II) exhibited distinct P1 domain signatures (S-G-G-G and G-G-G-G, respectively), consistent with monocot HKT classifications [17]. This dual presence of class I and II HKTs is a common feature in monocots, facilitating complementary functions in ion homeostasis. For instance, in barley (Hordeum vulgare), HvHKT1;5 (a class I transporter) plays a critical role in retrieving Na^+^ from the xylem, thereby protecting leaves from sodium toxicity [19]. The functional analogy between *HvHKT1;5* and our root-expressed *LpHKT1b/c* is intriguing and warrants further investigation. Similarly, the class II transporter LpHKT2 shares structural and phylogenetic similarity with OsHKT2;1 from rice, which mediates Na^+^/K^+^ symport under potassium starvation [18]. Notably, LpHKT3 clustered with OsHKT5 from rice, defining a divergent subfamily (group3). Its structural divergence (P1/P2 domains) implies possible functional specialization. Although its P3 and P4 domains were conserved, the divergent P1 and P2 domains suggest unique ion selectivity or regulatory mechanisms. This structural peculiarity is rarely reported in other grass HKT families and implies that LpHKT3 may have adopted non-canonical roles, possibly as a regulatory subunit or in a stress signaling pathway, rather than functioning as a typical ion transporter. This discovery expands the known diversity of the *HKT* gene family and highlights the unique evolutionary path of this gene in *L. perenne*. Similar divergence has been reported in *Brassica HKT* genes [1], but the evolutionary significance of *LpHKT3* requires functional validation. *LpHKT2’*s complex structure (11 exons) contrasts with the simpler architectures of other members (1–4 exons), potentially reflecting its role in Na^+^/K^+^ symport. In rice, alternative splicing of *OsHKT1;4* has been shown to regulate Na^+^ accumulation [7].

### 4.2. Dynamic Expression and Functional Roles of HKT Genes Under Salt Stress

*LpHKT* genes are highly expressed in roots and are upregulated at 6–12 hpt, may contribute to sodium sequestration via xylem unloading, potentially limiting Na^+^ translocation to shoots. The transient upregulation of *LpHKT1b/c* and *LpHKT2* at 6–12 hpt may reflect an early adaptive response to ionic imbalance, potentially facilitating rapid Na^+^ sequestration, suggesting a potential role in early ionic adjustment. In *Arabidopsis*, *AtHKT1;1* activity is regulated by extracellular Na^+^ accumulation [3,4,5,6]. The class II transporter *LpHKT2* is likely primarily involved in Na^+^/K^+^ symport activity. This inference is supported by the rapid accumulation of Na^+^ ions during the initial phase of treatment, contrasted with relatively minor fluctuations in K^+^ levels, and the observed pronounced decrease in the K^+^/Na^+^ ratio post-treatment. These findings suggest that intracellular Na^+^ accumulates rapidly, while K^+^ homeostasis is maintained more gradually. A previous report demonstrated that *AtHKT1;1* in *Arabidopsis* is upregulated under Na^+^ concentrations below 30 mM, whereas this upregulation is abolished or even reversed at higher concentrations [6]. In alignment with this observation, our study revealed a similar regulatory pattern: treatment with 25 mM NaCl induced significant upregulation of *LpHKT1b*, *LpHKT1c*, and *LpHKT2* across multiple tissues. However, under 100 mM NaCl treatment, the expression changes in these genes were markedly attenuated or statistically insignificant in most tissues. This dose-dependent response highlights a conserved regulatory mechanism by which HKTs contribute to ion homeostasis under varying levels of salt stress. While 25 mM and 100 mM NaCl effectively distinguish moderate vs. severe stress responses, future studies should employ graded salinity (e.g., 0–150 mM) to precisely quantify gene activation thresholds.

### 4.3. Promoter Cis-Elements and Multi-Stress Responsiveness

All *LpHKT* promoters contain stress-responsive *cis*-elements such as ABRE, MYB/MYC binding sites, and TC-rich repeats, suggesting that their expression is regulated by ABA signaling, drought, and oxidative stress. For example, ABRE motifs may mediate ABA-dependent salt stress responses, as demonstrated in *Arabidopsis*, where ABSCISIC ACID INSENSITIVE 4 (ABI4) binds to the ABRE motif of *AtHKT1;1* to modulate salt tolerance [38]. Additionally, the enrichment of GARE (gibberellin-responsive element) motifs in the promoters of several *LpHKT* genes suggests potential gibberellin-mediated regulation, consistent with reports that gibberellins antagonize salt stress responses [39]. These findings offer important insights into the cross-stress regulatory networks of *HKT* genes, highlighting their role as integrative nodes in coordinating responses to diverse environmental challenges.

Although this study inferred the functional roles of *HKT* genes through expression profiling and ion accumulation data, direct validation of their transport activity remains lacking. Critical functional validation in further study will be essential to conclusively determine the physiological roles of LpHKT1b, LpHKT1c, and LpHKT2 in sodium transport. Furthermore, we recognize that salinity tolerance is a polygenic trait likely involving allelic variations in multiple loci beyond the *HKT* gene family. Additionally, the experimental focus on short-term responses (within 24 h) limits insights into chronic stress adaptation. Future studies extending treatment durations and incorporating phenotypic analyses (e.g., biomass, photosynthesis) will clarify the long-term roles of *HKT* genes. Notably, the truncated TrkH domain and shortened polypeptide chain (257 amino acids) of LpHKT3 suggest its potential role as a regulatory subunit or its involvement in non-canonical pathways, warranting further investigation through functional assays.

## 5. Conclusions

In summary, this study provides the first genome-wide identification and characterization of the *HKT* gene family in perennial ryegrass (*L. perenne*). We delineated five *LpHKT* genes into three phylogenetically distinct subfamilies, uncovering a novel, structurally divergent member, *LpHKT3*. Promoter analysis indicated that these genes are likely regulated by multiple stress signaling pathways. Furthermore, the salt-responsive expression patterns of *LpHKT1b*, *LpHKT1c*, and *LpHKT2*, coupled with the observed ion homeostasis dynamics in salt-tolerant cultivars, strongly suggest their pivotal role in mediating Na^+^/K^+^ balance under salinity stress. These findings not only deepen our understanding of salt tolerance mechanisms in forage grasses but also establish a valuable genetic resource for future functional studies and the molecular breeding of improved ryegrass varieties with enhanced resilience to abiotic stresses.

## Figures and Tables

**Table 1:** HKT gene family members in *L. perenne*.

Gene ID	Name	Length (aa)	Mw (kDa)	pI	TrkH Domian
SNK15_009548	*LpHKT1a*	404	43.7	8.6	196–404
SNK15_009550	*LpHKT1b*	504	55.2	9.95	214–421
SNK15_032959	*LpHKT1c*	499	55.1	8.47	150–489
SNK15_037153	*LpHKT2*	1278	142.1	9.06	239–451; 492–537; 594–779; 936–1148; 1187–1236
SNK15_009547	*LpHKT3*	257	28.5	8.18	190–257

aa: amino acid residues; pI: isoelectric point; TrkH: conserved transporter domain.

## Data Availability

The original contributions presented in this study are included in the article/Supplementary Materials. Further inquiries can be directed to the corresponding authors.

## Supplementary Materials

The following supporting information can be downloaded at: https://www.mdpi.com/article/10.3390/genes16111341/s1, Table S1: The primers list; Table S2: Protein-coding potential.
