# Supplementary material for: Identification of the High-Affinity Potassium Transporter Gene Family in Perennial Ryegrass (Lolium perenne) and Its Potential Role in Salt Stress
Source: Genes (Basel). 2025 Nov 7;16(11):1341. doi: 10.3390/genes16111341 (PMC12652100; doi:10.3390/genes16111341)
Supplement: Supplementary file 1 [file genes-16-01341-s001.zip › genes-3894233-supplementary.pdf]

Supplementary Materials

Table S1. The primers list.

Gene_ID	Gene Name	forward primers (5'-3')	reverse primers (5'-3')	purpose
**SNK15_009550**	*LpHKT1b*	CATCTCCCAACACGTCCAC	TGGATATTGCCTTGTCGAGC	qRT-PCR
**SNK15_032959**	*LpHKT1c*	TTTCGAGCATGGTCTCAGTG	TTGACGTAGGTGAAGTGCAG	qRT-PCR
**SNK15_037153**	*LpHKT2*	GTGGTCTTTGGGTACTTCTCTG	GATGCCTTTCTTGTTCAGTGG	qRT-PCR
	LpEF1a	GGCTGATTGTGCTGTGCTTA	CTCACTCCAAGGGTGAAAGC	qRT-PCR
	LpHKT1c	TTTCGAGCATGGTCTCAGTG	TTGACGTAGGTGAAGTGCAG	in situ hybridization
	18S	GGTAATTCCAGCTCCAAT-	GTTTATGGTTGAGACTAG	in situ hybridization
	18sRT	GTTTCAGCCTTGCGACCATACT		in situ hybridization

Table S2. Protein-coding potential.

ID	C/NC	CODING POTENTIAL SCORE
LpHKT3	coding	1.60001
LpHKT1a	coding	3.76757
LpHKT1b	coding	4.94073
LpHKT1c	coding	5.75278
LpHKT2	coding	9.32915
